# Return on investment from quality improvement programmes in mental healthcare; a Delphi study on conceptual uncertainty and ambiguity

**DOI:** 10.1186/s12913-025-13484-0

**Published:** 2025-10-08

**Authors:** S’thembile Thusini, Tayana Soukup, Claire Henderson

**Affiliations:** 1 Health Services and Population Research Department, Institute of Psychiatry, Psychology and Neuroscience, King’s College London, London, UK; 2 Department of Surgery and Cancer, Faculty of Medicine, Imperial College London, London, UK

**Keywords:** ROI, Return on investment, QI, Quality improvement, Consensus, Dissensus

## Abstract

**Introduction:**

Return on Investment (ROI) is a recommended and thus legitimatised approach to assessing the value of healthcare programmes, including in Quality Improvement (QI). In its accounting origins, ROI estimates monetary benefits against investments. In health economics, ROI estimates a monetary value of healthcare related benefits against costs. In a recent single site study, we explored the metaphorical use of ROI as a concept of benefits from QI in-order to develop a QI-ROI conceptual framework. We found that in QI, both monetary and non-monetary benefits are deemed as legitimate ROI by mental healthcare leaders. However, various ambiguities and uncertainties associated with QI-ROI conceptualisation were also apparent. As such, in the current study, we explored consensus on the QI-ROI concept with a wider group of mental healthcare leaders across multiple sites. We then assessed the potential impact of the consensus levels on the stability of our QI-ROI conceptual framework.

**Methods:**

We ran two rounds of Delphi through Qualtrics online platform. Public sector mental health leaders were approached UK-wide (N = > 100). Only leaders from England participated (*N* = 23). This included board members (*n* = 15), directors (*n* = 2), and QI leaders (*n* = 6). Sixty-seven items were rated, including patient outcomes, development (e.g., culture), external benefits (e.g., socio-economic), incentives (e.g., reputation), implementation outcomes (e.g., sustainability), and monetised benefits. Consensus was measured using interquartile range and median. We also collected qualitative data to help explain participant responses.

**Results:**

There was consensus on 45 of 67 (67%) of the items in our existing QI-ROI conceptual framework. There was dissensus on 22 of 67 (33%) items. Positive consensus (item acceptance) was on 33 of 45 (73.3%), negative consensus (item rejection) was on 5 of 45 (11.1%) items, and indecision was on 7 of 45 (15.6%). Patient outcomes were rated as most relevant, followed by developmental benefits, and benefits for external stakeholders. External incentives and monetised outcomes were rated as least relevant and legitimate as QI-ROI. Areas of indecision and dissensus were largely on implementation outcomes, measurement, and monetisation of QI benefits. Qualitative data indicated that indecision and dissensus was likely related to organisational needs, or the novelty of some related benefits.

**Conclusion:**

Mental healthcare leaders value various QI benefits depending on organisational needs. Thus, although there is consensus on most aspects, some ambiguity and uncertainty may remain. Regardless, QI-ROI is viewed as both monetary and non-monetary benefits. This has implications for similar organisations and policies relating to the assessment of the value of QI.

**Supplementary Information:**

The online version contains supplementary material available at 10.1186/s12913-025-13484-0.

## Introduction

A number of institutions such as the World Health Organisation [1] recommend Return on Investment (ROI) as a tool to assess the value of healthcare programmes. This legitimises ROI for programmes such as Quality Improvement (QI) [2, 3]. As such, healthcare organisations are increasingly advised to measure ROI [1, 4]. However, some within and outside healthcare have raised concerns that the adoption of ROI may be at the expense of broader value [5, 6]. Thus, ROI as applied in QI programmes (QI-ROI) must be clarified. In its accounting origins, ROI measures the performance of monetary investments over time [7]. The output is often presented as profit. In health economics, ROI estimates monetary value of healthcare related benefits against their costs [2], the output often presented as cost or efficiency savings [8].

ROI can be used to estimate monetary value before or after programme implementation [2, 9]. Unlike small projects, QI programmes are large-scale interventions that aim to improve multi-level issues that ultimately impact patient outcomes [10]. QI initially aimed to solve local quality issues but later sought to achieve transferable research outputs beyond local settings through Improvement Science [11]. Related to QI and Improvement Science is Implementation Science which promotes the adoption of interventions through dedicated strategies, in search of positive implementation outcomes e.g., sustainability [12, 13]. As such, QI benefits may be diffuse. Further, mental healthcare favours outcomes such as reduced stigma [14]. Such outcomes including those from QI programmes may not be readily measurable and monetisable [3]. Unlike cost-benefit analysis, ROI often takes a programme’s investors perspective rather than a population perspective [4]. At an organisational level, healthcare leaders invest in QI.

This study was part of a larger project which aimed to develop a QI-ROI conceptual framework for application in QI programmes within mental healthcare and other healthcare disciplines. As such, the current study sought to refine the framework under our development. Our recent single site based in a United Kingdom (UK) National Health Service (NHS) mental healthcare organisation indicated that QI-ROI is conceptualised as any benefit that contributes to local strategic goals. This study also indicated multiple ambiguous expectations from QI, and uncertainties over QI benefits [15]. Specifically, various internal and external benefits were deemed part of an organisation’s QI-ROI. We also identified some apprehension over benefit monetisation; QI benefits with or without monetisation were seen as of value in and of themselves [15]. As such, monetisation was seen to limit the perception of QI value, and go against health and social care values that champion intangible benefits such as patient experience and social justice [15]. The implications of these findings are important to consider.

Firstly, ambiguity may force compromises to satisfy different organisational stakeholders. For example, whether to prioritise internal or external benefits, and which internal/external benefits to prioritise. Secondly, healthcare leaders have an obligation towards financial governance. This drives expectations to monetise benefits as credible evidence of efficiency savings [8]. Thirdly, uncertainty as a result of lack of concrete evidence of benefits is likely to encourage controversial value judgements over QI value. Thus, as ROI may increasingly be used to assess the value of QI programmes, it is crucial to explore the broader consensus on the views raised by our previous findings. Clarity here may help assess the stability of the QI-ROI concept as we have developed it, to bring it towards maturity and enhance its operationalisability [16].

### Aims

Our aim was to explore consensus on the relevance, legitimacy and eligibility of QI benefits as part of our QI-ROI conceptual framework in the context of a mental healthcare institution.

## Methods

### Study setting

This study involved leaders from multiple UK public sector mental healthcare organisations. However, QI applies similarly across disciplines. NHS mental healthcare organisations are known as NHS Trusts that provide health and social care services for persons with acute and chronic mental health disorders. As such, we adopted an institutional approach. Institutions are perceived as collections of shared stable rules, meanings and interpretations used by a legitimised social group [17]. Thus, the institutional focus enhanced our understanding of the prevailing concept of QI-ROI beyond a single mental health Trust, with potential implications for similar organisations. The study was conducted online between December 2022 and April 2023. The study was registered via King’s College London research ethics committee, registration: MRSP-22/23-33873. Our study checklist is supplied as a Supplementary file 1.

### Participants

The inclusion criteria were the NHS Trust mental healthcare leaders with knowledge of QI and ROI. We excluded leaders who were not in an influential decision-making position about either QI or ROI from QI. Based on our previous study [15], participants’ familiarity to ROI largely reflected lay or novices as mainly related to their awareness or familiarity linked with role e.g., if asked to report on or perform ROI analysis or may have had discussions about it at the leadership level. As such, we deemed ROI as applied to healthcare QI as novel. Therefore, we did not assess levels of expertise of participants. Instead, participants were asked to state their years’ experience in role, ROI and economic evaluations as a measure of their familiarity with ROI, QI investment and evaluation. This is in-line with seeking answers to value questions where judgements but not technical expertise about competing social goals are crucial [18].

We employed purposive and snowballing sampling techniques. Firstly, participants from our preceding qualitative study [15] were invited. Secondly, new participants were identified through organisations’ websites and social media. Lastly, potential participants were asked to help identify new contacts. Individuals were approached in all four UK countries (England, Wales, Scotland, Ireland). Participants were supplied with information sheets and given opportunities to ask questions at least two weeks before the study. Written consent was obtained prior to completing the survey.

### Study design

**Fig. 1:**
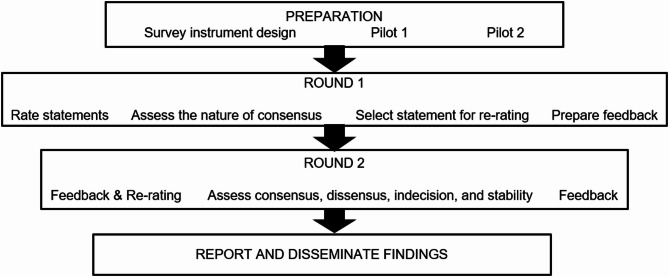
Delphi flowchart

The study employed a Delphi approach which incorporated qualitative comments to aid explanations to quantitative findings. Specifically, we took a policy Delphi approach where different views are explored for deeper insights on an issue, beyond merely measuring or building consensus [19]. Such a Delphi may allow individuals to negotiate reality, or raise awareness about other meanings of a phenomenon [19, 20]. The outcomes of this may be better clarity on views, making way for consensus on a shared meaning or informal agreements over time [19, 20]. As such, we explored the existence and nature of both consensus and dissensus. Figure 1 illustrates our steps in preparation, implementation and evaluation of the Delphi.

### The Delphi strategy

We ran two Delphi rounds to minimise attrition and because we had already identified items to be rated based on themes from a previous study [15]. The first-round items were based on these four themes: [1] organisational performance (patient and financial benefits) [2], organisational development (e.g., culture) [3], external incentives (e.g., reputation), and [4] implementation outcomes (e.g., sustainability). We selected items that we deemed would enable comparison of benefits within and between themes. We then divided items into two sections: Section A to assess item relevance and Section B to assess item legitimacy and eligibility (Table 1).

Item relevance was assessed on patient outcomes, financial benefits, organisational development, and external incentives. Items were listed randomly to encourage participants to evaluate them individually rather than as a group. Item eligibility and legitimacy was assessed on the most uncertain aspects from the previous study. These were intended versus unintended benefits, short versus long-term benefits, implementation outcomes, external benefits, measurable, attributable, and monetisable benefits, and legitimacy of monetisation. The survey was piloted twice with two QI leaders and a health economist who were not part of the study.

### Data collection

Data collection was carried out via secure and anonymised Qualtrics software [21], using two Likert-type scales [22]. A 10-point scale was used in Section A (benefit relevance). Participants rated items based on how relevant they thought each presented statement was to return on investment from a large-scale QI programme from 1 (least relevant) to 10 (most relevant). In Section B we ascertained benefit eligibility and legitimacy as part of QI-ROI using a 7-point scale. In this section, participants rated their level of agreement from 1 (strongly disagree) to 7 (strongly agree). In total, there were 67 items presented in the survey – of this, 26 items are presented in the first section (relevance) and 41 items are presented in the second section (eligibility). To avoid missing data, participants were required to provide a response to all statements before progressing. Our Qualtrics survey is available as Supplementary file 2.

### Criteria for consensus and dissensus

We used the interquartile range (IQR) as a measure of spread to determine the strength of consensus, and the median (Mdn) as a measure of central tendency for the location of consensus [18]. Given that no single guidance exists for IQR thresholds to determine consensus in Delphis, we based our criteria on multiple sources [18, 23]. Based on these sources, we noted that an IQR at or near midpoint may denote moderate consensus, indecision or dissensus depending on the size of a spread of views. Based on the commonly used thresholds, we pre-determined that an IQR of ≤ 2 denotes consensus on both 7 and 10-point scales, whilst an IQR of ≤ 3 may be acceptable on a 10-point scale if the IQR does not cross the mid-point (combine negative and positive views).

We noted that any consensus based on IQR 3 (10-point scale), denotes a low level of variation of views and hence deemed moderate (or not strong). We then graded consensus strength as follows; IQR ≤ 1 = very strong, IQR ≥ 1 to ≤ 2 = strong, IQR ≥ 2 to ≤ 3 = moderate. For neutrality or indecision, we noted midscale points. On a 7-point scale, items from medians 4 to 5 inclusive were deemed to be at midscale, whilst on a 10-point scale, item medians from 5 to 6 inclusive were deemed mid-scale. Thus, a narrow IQR at mid-scale of any scale was viewed as undecided or neutral, e.g., IQR 2, medians 5–6 on a 10-point scale OR median 4 and IQR 1 on a 7-point scale are neutral. Dissensus was deemed to exist where wide IQRs (> 3) and extreme outliers existed. An IQR > 3 on a 7-point scale, and > 4 on any scale denoted significant dissensus.

To determine outliers, we used the Tukey boxplots [24]. Some outliers may be very close to a median, therefore not extreme, (e.g., Mdn 6, outlier 7). Other outliers may be on the same side, and may not be considered variations of positive or negative views. To minimise participant workload and focus on areas of significant dissensus, where the boxplots indicated the presence of an outlier on an item, we accepted outliers as true if > 2 scores from a median. Items indicating indecision and dissensus, were re-rated, and assessed for stability. To determine group stability, we measured the variation of the relative interquartile range (RIR), as a percentage point difference in RIR between rounds. RIR variation cut-offs can range from 15% to 50% [23, 25]. We deemed an RIR variation of > 0.3 (30%) as significant instability [25] to help us note potentially meaningful instability in views and thus our conceptual framework.

### Feedback

Post round 1, items for re-rating were sent to participants with feedback in the form of boxplots with group medians per item. We also added associated comments on the relevance, eligibility, and legitimacy of associated items indicated by the results. Participants were asked to agree or disagree with the feedback and provide comments to explain their views on the results. As seen in Supplementary file 3. Feedback post round 2 was given as a summary of significant changes.

### Data analysis

We performed descriptive analysis using the IBM SPSS version 28 [26]. Descriptive data included minimum and maximum score, Q1, median (Mdn), Q3, and IQR. Boxplots were also downloaded to be examined for outliers. The relative interquartile range (RIR) variation for re-rated items was calculated by hand as RIR = Q3-Q1/median, and the difference in RIR from round 1 and 2 as the variation [25]. Qualitative data from both rounds were assessed for any additional data that can support, confirm, or refute quantitative data.

**Table 1:** List Delphi statements by section

Section A items: Relevance	Section B items: Eligibility	
**Patient outcomes** 1. Health outcomes 2. Population health 11. Access to care 17. Patient outcomes e.g., patient experience 26. Preventing patient mental health crises **Financial outcomes** 3. Cost-saving 6. Financial sustainability 10. Revenue generation 22. Avoiding costly care 23. Profit generation **Organisational development** 4. Capability development 5. Capacity development 8. Productivity 9. Efficiency 12. Staff outcomes e.g., staff experience 13. Internal collaboration 14. Quality and safety culture 19. Research development 20. Innovation development 21. Organisational sustainability **External incentives** 7. Reputation for quality care 15. Registration status e.g., being Foundation Trust 16. Oversight benefits e.g., improved CQC rating 18. Provider of choice 24. Competitiveness 25. External collaboration	**Intervention outcomes** 27. Only intended goals represent QI-ROI 28. Benefits beyond goals (e.g., unintended developments) 29. Lessons learnt (e.g., from failed QI) 30. QI legacy (e.g., raised safety awareness) 31. QI cannot fail as it trial and error 32. QI failed if not goals not achieved **Implementation outcomes** 33. QI failed if programme not spread 34. QI failed if new practice not embedded 35. QI failed if programme/benefits not sustained 36. Problem solving/programme speed is a sign of QI embedment 37. Problem solving/programme speed is an indicator of QI-ROI **Timing of ROI assessment** 38. Short-term outcomes 39. Long-term outcomes 40. Both short and long-term outcomes are part of ROI **External benefits** 41. Service-user socio-economic benefits 42. Friends, families, carers’ benefits 43. External partners benefits 44. Community and societal benefits **Measurable benefits** 45. Only measurable benefits are ROI 46. Immeasurable benefits are equally valid ROI	**Measurable benefits (cont.)** 47. Immeasurable benefits are more valid as ROI 48. Immeasurable benefits are sometimes more valid as ROI 52. Difficult to monetise benefits are sometimes more important **Monetisable benefits** 49. Only monetisable benefits are ROI 50. Difficult to monetise benefits are equally valid as ROI 51. Difficult to monetise benefits are more important **Monetisation legitimacy** 53. Monetisation is valid as it is the stipulated requirement 54. Monetisation is valid as it is the best practice 55. Monetisation is impractical, there should be an alternative 56. Monetisation is against professional values 57. Monetisation is against mental healthcare values **Benefit attribution** 58. Only benefits that be directly linked to a programme are ROI **Benefit evidence** 59. Valid indicators of hard to measure benefits are valid evidence 60. A narrative report of benefits is valid evidence for ROI 61. Subjective judgement of benefit measurement is acceptable 62. Subjective judgement is valid evidence of ROI if criteria agreed 63. Subjective criteria should be decided per Trust 64. Subjective criteria should apply across mental healthcare Trusts 65. Financial proxies are acceptable as ROI evidence 66. A narrative report of difficult to monetise benefits is acceptable 67. Subjective judgement about monetary benefit is valid evidence

## Results

Of the 100 approached contacts, 56 (56%) were interested in the study. Twenty-three (41%) of those took part in both Delphi rounds. Although a large interest in the study was generated, most leaders were unable to partake, some due to work pressures at the time of the study e.g., disruptions due to frontline staff pay dispute strikes, whilst some leaders reported being too busy. One potential participant was excluded as they were QI lead in a physical health Trust (not mental healthcare). Participants on both rounds were all from the English NHS (*N* = 23). They were executive board members (*n* = 9), non-executive (*n* = 3), QI and board directors (*n* = 3), QI lead/director (*n* = 6), clinical directors (*n* = 2). The group had an average of 5–10 years’ experience in their role, and 5–10 years’ experience in ROI and economic evaluation of QI. Each survey took 10–15 min on average, although some were not completed at once.

### Round 1

In round one, 62 of 67 (92%) items achieved an IQR of ≤ 3, our maximum cut-off for consensus. Although this indicated a high level of consensus as per IQR, 22 of 62 (33%) some items had IQR of 3 and or outliers. Removing these items left 45 of 67 (67%) items where consensus was found, and seven (10%) items that indicated indecision. Once outliers, dissensus and indecision were determined, clear positive and negative consensus was deemed to have been achieved on 37 of 67 (55%) items. Therefore, 30 of 67 (45%) items remained for re-rating in round 2.

### Round 2

In round 2, there were missing data points from responses of three participants. This amounted to 10 missing data points in total. Qualtrics indicated that those surveys had not been completed by their assigned period of seven days, although they could not rule out technical interference. Therefore, the missing data were deemed likely to be missing completely at random. As the pattern of missing data appeared unlikely to change the outcome of the study, we proceeded with the analysis without replacing these data points [27].

Based on our analysis, five of 30 (16.7%) re-rated items exceeded our RIR (relative interquartile range) cut-off of < 30%, thereby indicating significant instability. Most instability was related to the change in the position of outliers; where there had been numerous outliers around narrow IQRs, views converged towards the median. This eliminated outliers, but increased IQRs to > 3. Thus, these items remained areas of dissensus. Overall, consensus after round 2 remained at 45 of 67 (67%) items, and dissensus remained on 22 of 67 (33%) items. Within consensus items, positive consensus (item acceptance) was on 33 of 45 (73.3%), negative consensus (item rejection) was on 5 of 45 (11.1%) items, and indecision was on 7 of 45 (15.6%). Supplementary file 4 illustrates results per item.

### Summative results: rounds 1 and 2

#### Section A: item relevance as QI-ROI

Results are reported per section; Relevance (26 items) and Eligibility (41 items) as outlined in Table 1. Amongst the factors covered by Relevance, patient outcomes were rated as most relevant to QI-ROI, followed by development, financial outcomes, and external incentives. Consensus here was moderate to high, with few outliers and areas of dissensus or indecision.

### Patient outcomes

Service user health outcomes received the highest rating and strongest consensus (IQR = 2, Mdn = 10). This was followed by population health (IQR = 2, Mdn = 8), service user experience (IQ = 3, Mdn = 9), and service user access to care (IQR = 3, Mdn = 8). In round 2, the IQR of access narrowed from 3 to 2 (RIR variation 0.13), indicating slight convergence on its high relevance to QI-ROI. There was dissensus on preventing mental health crises (IQR = 4, Mdn = 7.5, RIR 0). This dissensus is explained in more detail in the qualitative feedback.

### Financial benefits

Financial sustainability received the highest rating (IQR = 3, Mdn = 8), followed by cost-avoidance (IQR = 3, Mdn = 7). There was consensus that profit generation was viewed as less relevant to QI-ROI (IQR = 3, Mdn = 3). There was a minor degree of indecision regarding cost-savings (IQR 2, Mdn 6), and regarding revenue generation (IQR = 3, Mdn = 4). The cost-savings IQR narrowed from 3 to 2 (RIR variation 0.2), indicating slight convergence on it as relevant to QI-ROI. However, its relevance remained low (6 of 10). The revenue generation RIR variation was 0, indicating no change in views that it is likely not seen as relevant to QI-ROI.

### Organisational development

Improvement of culture received the highest rating and consensus (IQR = 2, Mdn = 9). This was closely followed by productivity (IQR = 1.5, Mdn = 8), staff outcomes, efficiency, improved capability, and organisational sustainability (IQR = 2, Mdn = 8). These were followed by improved capacity, innovation, internal and external collaboration (IQR = 2, Mdn = 7). External collaboration lost outliers but acquired a slightly broader IQR from 1.5 to 2 (RIR variation 0). This indicated stability in external collaboration as being of moderate relevance. There was some dissensus on development of research skills as relevant (IQR = 2, Mdn = 6, one outlier). In round 2, the research item IQR narrowed from 2.5 to 2 (RIR variation 0). This suggested stability on research item as of lower relevance to QI-ROI, albeit potential dissensus.

### External incentives

Within this group, the reputation for quality care was given the highest score (IQR = 3, Mdn = 8), followed by oversight benefits (IQR = 3, Mdn = 7). Areas of dissensus were improved status (IQR = 3, Mdn = 4), being a provider of choice (IQR = 5, Mdn = 6.5), both with RIR variations 0. Competitive advantage (IQR = 4, Mdn = 5.5), with RIR variation 0.78, indicating significant instability due to a narrowing of IQR from 6 to 4. Nonetheless, this indicated low relevance for these items as per medians, albeit with some significant dissensus as indicated by wide IQRs.

#### Section B: QI outcome legitimacy and eligibility as QI-ROI

The items in this section were measured using a 7-point scale. Most items achieved IQRs of 0 to 1. However, there were multiple outliers on several items, and thus several items were re-rated. Overall, benefits beyond intended QI goals, as well as benefits that are difficult to measure, monetise, and attribute received higher scores.

### Intended versus unintended outcomes

Highest rating and strongest consensus was achieved for benefits beyond intended programme goals (IQR = 0, Mdn = 6), including lessons from QI programmes (IQR = 1, Mdn = 6), legacy left by QI programmes (IQR = I, Mdn = 6). There was negative consensus on the statement that QI cannot fail (IQR = 2, Mdn = 3), meaning this statement was rejected. There was dissensus that QI failed if not goals not achieved with Mdn = 3, but this was unstable with RIR = 0.7.

### Short versus long-term benefits

The item that combined short and long-term benefits are part of QI-ROI achieved the strongest consensus and highest rating (IQR = 0, Mdn = 6). Negative consensus and low scores were short-term only (IQR = 3, Mdn = 3), and long-term only (IQR = 2, Mdn = 3). The RIR variation of short outcomes alone was 1, due to a widening of IQR from 0 to 3. This indicated significant instability on only short benefits alone are eligible as part of QI-ROI. Long-term outcomes’ IQR increased from 1 to 2, RIR variation 0.17, indicating a slight instability. Thus, there was movement in the short-term outcome item. In the absence of the combined impact item in the second round, participants appeared to favour either short-term or long-term outcomes.

### Implementation outcomes

There was high dissensus on implementation outcomes with wide IQRs and outliers. Spread achieved IQR = 3, Mdn = 4, embedding IQR = 4, Mdn = 5, sustainability IQR = 2, Mdn = 5. There was significant instability with the item on embedding QI with RIR variation at 0.5, and slight instability on the item sustainability with RIR variation at 0.3. This was due to the widening of the IQRs, which replaced multiple outliers. Speed of implementation received the highest score and strongest consensus (IQR = 1, Mdn = 6).

### External benefits

Benefits for friends and family achieved strong consensus and high rating (IQR = 1, Mdn = 6). Also highly rated with strong consensus were benefits to community and society (IQR = 0.5, Mdn = 6), and benefits for external partners (IQR = 1, Mdn = 6). There was an outlier for service user socio-economic (IQR = 0, Mdn = 5, RIR variation 0), indicating minor dissensus.

### Measurable and monetisable benefits

There was consensus that immeasurable and non-monetisable are equally valid as measurable and monetisable benefits; immeasurable (IQR = 2, Mdn = 5), non-monetisable (IQR = 1, Mdn = 5). There was consensus that immeasurable and non-monetisable benefits are sometimes more valid; immeasurable (IQR = 2, Mdn = 5) non-monetisable (IQR = 1, Mdn = 5.25). Finally, there was consensus that immeasurable benefits are *not* more valid as QI-ROI (IQR = 2, Mdn = 3). There was a level of indecision that non-monetisable are *not* more valid than monetisable ones (IQR = 2, Mdn = 4, relative RIR 0), indicating some indecision around these items.

### Legitimacy of monetisation

There was consensus that monetisation is not against their professional values (IQR = 1.5, Mdn 2) and mental healthcare values (IQR = 2, Mdn = 2). There was an indication of a group indecision about whether monetisation of QI benefits was viewed as a requirement (IQR = 1.5, Mdn = 4), best practice (IQR = 2, Mdn = 4), or as impractical (IQR = 2, Mdn = 4). Altogether, this indicated an openness and or an indecision about the legitimacy of monetisation.

### Attribution versus contribution

There was rejection of attributable benefits as the only evidence of QI-ROI (IQR = 3, Mdn = 3). Subjective judgement alone was not accepted as valid evidence of QI-ROI (IQR = 2, Mdn = 3). Subjective judgement was however accepted if there were set criteria, albeit, slightly unstable (IQR = 3, Mdn = 5, RIR = 0.4). There was indecision about whether criteria would best be set for each Trust (IQR = 1.5, Mdn = 4), or the whole mental healthcare (IQR = 2, Mdn = 4).

There was consensus that narrative reports, financial proxies, and indicators were acceptable evidence of QI-ROI; narrative reports (IQR = 1, Mdn = 6), proxies (IQR = 1, Mdn = 5), and indicators (IQR = 1, Mdn = 5,5), agreed judgement criteria (IQR = 1, Mdn = 5). However, there were two areas of instability due to widened IQRs. These were narrative reports as acceptable evidence of ROI (RIR variation 0.13), and subjective judgement as valid evidence of ROI if criteria agreed (RIR variation 0.4). The latter indicated significant instability of the item.

### Rounds 1 and 2: qualitative analysis

In round 1, only three of 23 participants entered comments. The comments entered did not indicate a need for changes in statements that were to be re-rated in round 2. These comments were added to round 2 comments for analysis. In round 2, 13 of 23 participants entered comments. Thus, qualitative data were assessed by hand for any additional information that clarified, supported, or disputed quantitative findings. We prepared qualitative data on word document to allow us to visualise the data and note any additional insights arising from qualitative comments. Comments ranged from one word to extensive comments. As such, it was not possible to develop themes.

Qualitative data supported quantitative findings that patient outcomes are seen as the most relevant. However, participants explained some of the ratings. For example, some emphasised an increasing need to focus on financial outcomes. Several participants saw financial outcomes as a positive but unintended consequence of improved care and processes. A participant described this in terms of primary (patient outcomes) and secondary (financial benefits). In this process, short-term and intended goals are seen as important early indicators of QI-ROI. However, long-term and unintended benefits were also seen as a legitimate part of QI-ROI.

Participants hinted at the factors that determined their conceptualisation of QI-ROI. For some, the current financial context behind the national frameworks influences needs to balance quality and financial outcomes. However, participants did not appear to focus on lower end variables like cost-saving and cost-avoidance. Instead, financial sustainability, a long-term variable, was most valued. This indicated a focus on impact rather that outputs or short-term outcomes in conceptualising QI-ROI, as illustrated by the following comment from one participant:

“In the current context, financial sustainability and resource implications needs to have equal weight in considering where to invest limited QI resource”. Participant 14; round 2

The views on financial sustainability as the most relevant financial outcome appeared to also be a result of an increasing systems view behind the QI-ROI concept. This view recognises the interconnectedness of mental healthcare. For example, some participants clarified that challenges such as preventing service users’ mental health crises may not be under the control of a single organisation. However, a participant commented that they did not see a connection between preventing crises and QI-ROI. This may indicate a lack of perceived connection to systems outcomes by some. Nonetheless, a systems perspective was used to explain and legitimate externalised outcomes as mandated by national agendas, as can be seen below.

“The current and relatively new ways of assessing business case looks very much at the wider socio-economic benefits and scores them. QI needs to follow the same model”. Participant 14; round 2

“The national business case model requires monetisation of benefits in the wider sense to support investments of public money - they have to reach a specific return score”. Participant 3; round 1

Participants clarified some of the low ratings. Market-based benefits such as profit were seen as not appropriate as healthcare aims to reinvest finances to support care provision. Further, intrinsic benefits like pride in own work are seen as more relevant than status or competitive advantage. A participant expressed that being a Foundation Trust (a semi-independent organisation) as an example of status benefits is no longer relevant. However, some had rated this statement high, based on the concept of status itself. Another participant stated that being provider of choice may also be meaningless as patient choice in NHS may not be possible.

In terms of oversight benefits, some clarified that although the regulator CQC (Care Quality Commission) is behind some improvements, it is not the only QI driver and focus of benefits. QI programmes are largely driven by internal rather than external agendas. Some participants indicated that they based their QI-ROI concept on their experience concerning the goals of QI. As such, some saw the current ROI philosophy as misaligned to how Trusts work. Therefore, there appeared to be conflict between internal and external QI agendas, as can be seen below.

“Whilst our funding expects us to be able to reduce costs, as an NHS mental health trust at present we are much more focused in our improvement work on ensuring care is safe and effective”. Participant 2, round 2

“Focus on improve care, this will lead to better outcomes, higher staff satisfaction which in turn will reduce costs - return on investment is approaching the subject from totally the wrong end”. Participant 16, round 2

“ROI needs to be seen a different in an NHS context than wider business return i.e., better than competitor isn’t a system benefit or return unless it leads to lost income coming back into the […] sector from another”. Participant 3, round 2

Two participants indicated that the neutral responses about the legitimacy of monetisation may be a result of ambiguity in the Delphi statement. Nonetheless, there were concerns about uncertainty over the evidence for QI-ROI. Participants stated that although narrative reports can aid understanding, the ability to quantify benefits was essential. This appeared to create dilemmas, as seen in how the challenges of obtaining evidence at times meant that some participants debated or excluded some benefits that were otherwise desired. This was also evident in the different perceptions on the legitimacy, feasibility of quantification and monetisation of valued benefits as seen below.

“The most relevant aspects are always the most difficult to measure”. Participant 2; round 1

“it is usually possible to monetise but the benefits may accrue to another party or part of the organisation”. Participant 3; round 2

“… it is not always possible to get a monetary benefit”. Participant 7; round 2

“Monetisation is how the wider public sector/Treasury assesses the impacts on the wider national economy this is how it has to work”. Participant 3; round 1

Professional judgement of QI benefits was accepted. However, participants were unclear whether organisation-specific or institutional criteria are best. Some participants indicated that they may have very specific goals for each programme. Nonetheless, participants indicated that core benefits such as health outcomes may be shared. Some participants were concerned that not insisting on evidence may prevent efforts to account for QI investments.

“It is too easy to say that we cannot quantify the financial benefits of QI projects and therefore agree a narrative evaluation is acceptable, however, this allows for a certain amount of complacency, and understanding of route cause issues and quantifiable problems to be agreed which is often the hardest part of QI work”. Participant, 5 round 2

“I very much agree the monetisation isn’t against any profession[al] value but the culture must change as we need to be able to prove the wider system benefits and thus, we need to use national tools in this respect”. Participant 14, round 2

Finally, participants agreed that unintended outcomes play a significant role in QI-ROI. Some commented that lessons are sometimes part of a QI programme, as supported by the trial-and-error philosophy. However, trial-and-error is seen as a means to reduce chances of failure, and not as a tool for poorly designed, wasteful programmes. Similarly, implementation outcomes such as spread, embedding, and sustainability may be intended, and thus part of QI-ROI. Participants emphasised that such outcomes can only be part of ROI where improvement has been shown. This implies a difference between a small QI projects and large QI programmes.

“…depends on whether the programme is designed to spread…a programme designed from the outset to spread could be viewed as a failure if it doesn’t”. Participant 2, round 2

## Discussion

Building on our previous findings [15], in this study, we found consensus on the relevance, eligibility, legitimacy of most QI outcomes as QI-ROI. Our current findings also indicated that precision on the QI-ROI concept may be difficult to achieve as there is potential for ongoing ambiguities and uncertainties over certain aspects within the framework. Nonetheless, there was consistency on ratings across Sections A (relevance), and B (legitimacy and eligibility). There was consensus on what appears to be core components of the QI-ROI concept, mainly improvement in service user benefits, organisational development and sustainability. Benefits to external stakeholders were also consistently highly valued. There was consistency between valuing comprehensive benefits and the views that both short and long-term outcomes are a measure of QI-ROI. Comparatively, monetary benefits were consistently seen as less relevant.

Financial sustainability and cost-avoidance were more preferred to cost-saving. Altogether, these financial benefits were seen as of moderate relevance to QI-ROI by many. However, these outcomes have been receiving increasing attention in healthcare [8]. This was also reflected in this study where the qualitative comments indicated that leaders are reflecting on a need to increase the status of financial benefits. Nonetheless, the secondary status of financial outcomes is in conflict with ROI traditions that favour market-based benefits. Market-based variables like profit, revenue generation, and competitive advantage were however rated highly by a few participants. Although uncommon in publicly funded healthcare, profit-making in the NHS can be found. This is associated with entrepreneurism to generate revenue in support for service provision [28]. Whether revenue and profit can be generated through QI is unknown.

External incentives like reputation, being provider of choice, competitive advantage are novel in UK publicly-funded healthcare. Competitive advantage embodies an organisation’s ability and capability to perform well beyond its competitors [29]. Competition was introduced in the UK healthcare to drive efficiency. Recently, QI methods have been employed to promote competitiveness [30]. It is yet unclear if QI can impact such variables. However, reputation for quality as an incentive was rated highly in this study. A good reputation may afford competitive advantage, improve status, and enhance oversight. A reputation for quality care may make for a provider of choice, thus increase market-share [31]. Whether users of public healthcare can actually exercise choice is unclear [32]. Nonetheless, our findings indicate that QI intervention and implementation outcomes may incentivise organisations in many ways internally.

There was some dissensus about implementation outcomes as part of QI-ROI. Implementation outcomes are also a relatively new focus, and may not be measured in many programmes [33]. Implementation insights enhance learning about programmes [33]. This may improve programme efficiency. Thus, both intervention and implementation benefits can help maximise returns. In-line with previous findings [4], the speed of problem identification and solution implementation received high positive consensus. Over time, proficiency in QI implementation may enhance work speed, productivity, efficiency, and sustainability. Some have attempted to measure this ROI of increased speed (e.g., as a result of reduced interruptions [34]). However, in complex contexts like healthcare, these benefits may be difficult to deduce and attribute [34].

Some of the valued in Section A (relevance) are difficult to measure and monetise e.g., patient experience. This was consistent with the high scores given to intangible benefits in Section B (legitimacy and eligibility). Intangible benefits are those that are difficult to measure, monetise, and attribute [17]. Although some QI benefits have established means of measurement, measurement is not always be feasible, valid, or reliable [17, 18]. According to Solid [3], this applies to most QI benefits. However, QI provides other benefits [3], although acknowledging them as QI-ROI would require local consensus on value judgements about such QI benefits.

Some dissensus and indecision may be related to the newness of rated concepts, which may change over time. For example, QI is not traditionally seen as research. However, as QI matures into Improvement Science, capabilities to generate data for use beyond the local context will be essential [11]. Further, although collaborating with different sectors is not new, formal integration is new, and will increase the relevance of system-wide benefits [8]. The need to contextualise or cater for multiple leadership obligations may also change views on QI-ROI.

Differences in world-views are inevitable due to ‘institutional complexity’ and dilemmas in interpreting differing demands [35]. Thus, concept instability as a result of ambiguity and uncertainty may be unavoidable, at least in the short-term. There may be various other explanations for differing views in the legitimacy, eligibility, and relevance of certain benefits as QI-ROI. These include professional backgrounds, QI programme goals, and organisational development needs. If and when core objectives have been achieved, organisations may then focus on saving costs [36]. The different foci may explain the overall stability of the consensus levels on core QI-ROI attributes, whilst the merits of others are debated. Institutional complexity thus means that consensus building is not a one-time event, but is rather emergent, with benefits (e.g., awareness) revealed over time through diverse feedback mechanisms [19].

### Policy and practice implications

These findings indicated that there might be a ‘hierarchy of organisational needs’ as an overriding explanation for the responses of healthcare leaders to ROI. Thus although, there are core benefits sought from QI, a ‘one size fits all’ QI-ROI concept may not exist. As such, flexibility may be needed to accommodate nuances within the QI-ROI framework. ROI may be perceived by some as a threat to QI investment or health and social care values [5]. Failure to acknowledge this may alienate some leaders or organisations from adopting ROI practice.

Healthcare is an institution that has its primary goal as the health (not the economy) of a society [37]. However, there is an increasing need to include financial accounting as part of QI governance [8]. Our findings indicate support for all these views. Predominantly, a willingness to compromise and elevate the status of financial benefits in healthcare QI exists. In contexts of ambiguity, uncertainty, multi-dependency, and constraints, various responses to institutional change are possible [38]. Where external agendas are misaligned with internal values and capabilities, negative responses such as manipulation may ensue [38]. Therefore, uncertainties, ambiguities, and conflicts must be explored as part of developing a usable QI-ROI concept.

### Strengths and limitations

Delphis have a potential for ‘forced consensus’ that can result from rephrasing statements between rounds [18]. We used two strategies to minimise this issue. Firstly, we created two statements to capture different perceptions of some variables. For example, to capture thoughts on cost-avoidance, one statement read ‘preventing costly care’, another ‘preventing mental health crises’. Although both may result in reduced costs, one is focused on cost, another on patients. Secondly, individual scores were not included as part of round 2 feedback; only group medians were supplied. Thus, participants blindly re-rated the items, based on their current views at the time against a group median, rather their individual previous scores. Although this is not normal Delphi practice, it may mean that participants viewed the questions with a fresh mind. This also provided an opportunity for checking potential conclusions with participants.

The study has some limitations. As the study only had two rounds, we were unable to ascertain sustained stability [21], however this is something that future research should explore further. Not all statements were re-rated in the second round, to minimise survey fatigue and encourage participants to focus on areas that needed more clarity. However, this meant stability was only assessed on 30 statements where there was indecision and dissensus. Therefore, it is not known if participants could have altered other scores over more Delphi rounds. Another potential limitation as pointed out by some participants, was that some statements may have been ambiguous. For example, different definitions of QI programmes. To reduce the survey size, the definitions of some terms e.g., QI programme had been provided in the first but not second round. However, re-issuing definitions may have minimised ambiguity. Nonetheless, some responses may reflect an issue with the format of the statements rather than participants’ views.

### Research recommendations

These findings reflect views of various organisational leaders. Future studies could run subgroup analysis to ascertain any differences by groups in perceiving QI-ROI, e.g., between QI leaders and board members This study also highlighted that participant may be conceptualising QI-ROI from both a project and programme perspective. It may thus be useful to study this distinction in future research to draw more differences between QI-ROI of small projects and large QI. Future research may also explore the contribution of implementation outcomes to QI-ROI. Researchers could explore face-face techniques such as the Nominal Group Technique and Concept Mapping to assess if better insights on QI-ROI could be gained through more interactive consensus exercises. Further, future study on the Delphi methodology may compare the effects of feedback using only group statistic rather than individual scores to ascertain which method may be more prone to social desirability or forced consensus.

## Conclusion

Undoubtedly, patient outcomes are core to any and all QI activity. Organisations also look to QI to develop and sustain them. In spite of ambiguities, there is coherence with health and social care values as central to QI-ROI in healthcare. In the current struggle for evidence of QI-ROI, defining acceptable benefits and their evidence must be negotiated with all relevant stakeholders. This means a view of QI-ROI that goes beyond financial measures as per traditional ROI. Prioritising one measurement philosophy over another risks creating inefficient blind-spots. Nonetheless, the QI-ROI concept needs boundaries to enable its operationalisation. This calls for attitudes that embrace challenges and innovative ways to articulate and not suppress QI value concepts in context. This includes supporting a system’s view, and tolerating ambiguity and uncertainty, whilst negotiating a shared view on QI-ROI.

## Supplementary Information

Below is the link to the electronic supplementary material.

Supplementary Material 1

## Data Availability

The datasets used and/or analysed during the current study are available from the corresponding author on reasonable request. Some data has been included in this published article as its supplementary documents.

## Abbreviations

QI: Quality Improvement
ROI: Return on Investment
QI-ROI: Return on Investment from Healthcare Quality Improvement
