# Supplementary material for: Return on investment from quality improvement programmes in mental healthcare; a Delphi study on conceptual uncertainty and ambiguity
Source: BMC Health Serv Res. 2025 Oct 8;25:1330. doi: 10.1186/s12913-025-13484-0 (PMC12505768; doi:10.1186/s12913-025-13484-0)
Supplement: Supplementary file 1 — Supplementary Material 1 [file 12913_2025_13484_MOESM1_ESM.pdf]

Supplementary file 1 The QI-ROI Delphi study checklist

ITEM AND DESCRPTION	PAGE NUMBER
TITLE AND ABSTRACT<br>The identification of a study as a Delphi process (1).	1
EPISTEMOLOGY<br>The positioning in a specific strand of theory of science (realist or constructivist), as well as formation of the objective and the statement of preliminary assumptions, e.g., about possible conflicting perspectives (1).	3<br>Lines 92-93
DELPHI VARIANT AND ROLE IN REASEARCH PROCESS<br>Statement and justification of which Delphi variant or which modification has been chosen and how they are situated in the research process (1).	3<br>Line 91
PURPOSE AND RATIONALE<br>The purpose of the study should be clearly defined and demonstrate the appropriateness of the use of the Delphi technique as a method to achieve the research aim. A rationale for the choice of the Delphi technique as the most suitable method needs to be provided (2).	2<br>Lines 57-68
FORMAL CONTEXT<br>This includes information on the research team conducting the project and other key information on the project background (e.g., funder or objective of the project context, study protocol, ethics) (1).	27<br>Lines 632-657
KNOWLEDGE BASE<br>This includes information on the consideration or integration of the current state of research i.e., the evidence base. It should also be indicated if the designated respondents are aware of it or how it can be ensured that they are aware of necessary contexts or are informed about them and in which social context they are located (1).	2

KNOWLEDGE AND KNOWLEDGE INTEGRATION<br>This concerns, in particular, the specifications of which kind of knowledge stocks and experiences are relevant and which perspectives area necessary to answer the respective research question, as well as how the different knowledge stocks are potentially weighted and these are to be bought together (1).	3<br>Lines 78-79
SAMPLE<br>Criteria for the selection of experts and transparent information on recruitment of the expert panel, sociodemographic details including information on expertise regarding the topic in question, (non)response and response rates over the ongoing iterations should be reported. The handling of autonomy should also be reported (1, 2).	3<br>Lines 78-79
DESCRIPTION OF METHODS<br>The methods employed need to be comprehensible; this includes information on preparatory steps (How was available evidence on the topic in question synthesised?), piloting of material and survey instruments, design of the survey instrument(s), the number and design of survey rounds, methods of data analysis, processing and synthesis of experts' responses to inform the subsequent survey round and methodological decisions taken by the research team throughout the process (2).	4-8
PROCEDURE<br>Procedure. Flow chart to illustrate the stages of the Delphi process, including a preparatory phase, the actual 'Delphi rounds', interim steps of data processing and analysis, and concluding steps	4
SURVEY INSTRUMENT<br>The survey instrument must be described in terms of its scope, structure, derivation, and testing of questions or items, the ratio of open and closed questions, integrated scale types, and graphic design (1).<br><br>DELPHI ROUNDS<br>This includes the number of Delphi rounds, as well as disclosure of the termination criterion (1).<br><br>DEFINITION OF CONSENSUS	4-6

Definition and attainment of consensus. It needs to be comprehensible to the reader how consensus was achieved throughout the process, including strategies to deal with non-consensus (2).<br><br>DEFINITION OF DISENSUS	
RESULTS<br>Reporting of results for each round separately is highly advisable in order to make the evolving of consensus over the rounds transparent. This includes figures showing the average group response, changes between rounds, as well as any modifications of the survey instrument such as deletion, addition or modification of survey items based on previous rounds (2).	9
FEEDBACK<br>Disclosure of statistical and graphic representation of feedback per round (1).	9
EVALUATION<br>This includes the definition of and dealing with consensus and disclosure of the quantitative and qualitative evaluation strategy, and also how these two aspects are weighted and combined (1).	19-23
QUALITY OF DATA AND INTERPRETATION<br>This includes reflection on the quality of the data collection and evaluation process. Quality criteria for quantitative or qualitative research should be applied depending on the epistemological positioning and the specific Delphi variant (1).	25
DISCUSSION AND LIMITATIONS OF FINDINGS<br>This refers to the critical reflection on the validity claim of the findings, a critical reflection of potential limitations and their impact of the resulting guidance depending on the epistemological basis (1, 2).<br><br>Reporting should include a critical reflection of potential limitations and their impact of the resulting guidance (2).	23-26

ADEQUACY OF CONCLUSIONS<br>The conclusions should adequately reflect the outcomes of the Delphi study with a view to the scope and applicability of the resulting practice guidance (1).	24
DISSEMINATION OF FINDINGS<br>Statement of how the findings will be processed or used beyond the Delphi study. The resulting guidance on good practice should be clearly identifiable from the publication, including recommendations for transfer into practice and implementation. A dissemination plan should include endorsement of the guidance by professional associations and health care authorities to facilitate implementation (1, 2).	27

1. Spranger J, Homberg A, Sonnberger M, Niederberger M. Reporting guidelines for Delphi techniques in health sciences: A methodological review. Zeitschrift für Evidenz, Fortbildung und Qualität im Gesundheitswesen. 2022;172:1-11.
2. Jünger S, Payne SA, Brine J, Radbruch L, Brearley SG. Guidance on Conducting and REporting DElphi Studies (CREDES) in palliative care: Recommendations based on a methodological systematic review. Palliative medicine. 2017;31(8):684-706.

Supplementary file 2: QI_Return on Investment Survey

Start of Block: Default Question Block

INTRODUCTION TITLE OF PROJECT:
The development of the QI-ROI conceptual framework for mental health; a DELPHI study. Ethical review reference: King’s College London; MRSP-22/23-33873

-------------------***-------------------

Thank you for taking part in this study

Please note that this study is about return on investment from using quality improvement (QI) methodologies in large-scale QI programmes. Large QI programmes are those designed to affect an entire organisation or a large part of an organisation, e.g., two or more departments.

This study aims to learn what mental healthcare leaders think about or mean by return on investment from large-scale QI. I would appreciate any further pertinent information you wish to highlight in the optional space at the end of each section.

There are 67 statements across two sections, and a final demographic question. Some statements have definitions for clarity. The survey should take about 10 minutes. You can go backwards and change your response if you wish.

Many thanks in advance.

S'thembile Thusini (Tay)
PhD student, King's College London

CONSENT FORM If **CONSENT** already **GIVEN**, please **go to end of page**, select I consent.

If not, please read carefully, select one option on the side and end page.

	PLEASE SELECT	
	YES (1)	NO (2)

1. I confirm that I have read and understood the information sheet dated 04/11/22 for the above project. (1)	O	O
2. I have had the opportunity to consider the information and asked questions which have been answered to my satisfaction. (21)	O	O
3. I understand that I must not take part if I fall under the exclusion criteria as detailed in the information sheet and explained to me by the researcher. (22)	O	O
4. I consent voluntarily to be a participant in this project. (24)	O	O
5. I consent to participate in this study via Qualtrics website. (25)	O	O
6. I understand that my identity will be concealed and that the Qualtrics website will not divulge my personal data. (26)	O	O
7. I understand that I can refuse to take part and can withdraw from the project at any time, without having to give a reason, up until the survey has been analysed. (27)	O	O

8. I consent to the processing of my personal information for the purposes explained to me in the Information Sheet. (28)	o	o
I understand that such information will be handled under the terms of UK data protection law, including the UK General Data Protection Regulation (UK GDPR) and the Data Protection Act 2018. (29)	o	o
9. I understand that my information may be subject to review by responsible individuals from King's College London for monitoring and audit purposes. (30)	o	o
10. I understand that confidentiality and anonymity will be maintained, and it will not be possible to identify me in any research publications. (31)	o	o
11. I agree that the research team may use my data for future research. This data will not be identifiable in any report. (32)	o	o
12. I understand that the information I have submitted will be published as a report and may be used in conferences and other presentations related to this study. (33)	o	o

13. I agree to be re-contacted in the future by King's College London researchers regarding this project entitled "The development of the QI-ROI conceptual framework; a DELPHI study. (34)	O	O
14. I agree that the researcher may retain my contact details so that I may be contacted in the future by King's College London researchers for other related research. (35)	O	O
15. I agree to be contacted in the future by King's College London(KCL) researchers who would like to invite me to participate in future studies of a similar nature. (36)	O	O
16. I consent to have my anonymised direct quotations used in this study's publications. (37)	O	O
17. I consent to members of KCL having access to my anonymised data. (38)	O	O
18. I wish to receive a copy of the final report via email. (39)	O	O

CONSENT FORM Please select one

O Yes I consent (1)

O No I do not consent (2)

Skip To: End of Survey If Please select one = No I do not consent

End of Block: Default Question Block

Start of Block: SECTION 1

Section A SECTION A: RELEVANCE

In my previous studies, a number of QI outcomes were seen as important benefits. The statements below represent some outcomes from those domains.

Please indicate how relevant you think each statement is to return on investment from a large-scale QI programme.

____

Q.1 1.

	Least relevant 1 (1)	2 (2)	3 (3)	4 (4)	5 (5)	6 (6)	7 (7)	8 (8)	9 (9)	Most relevant 10 (10)
Improved service user health outcomes (1)	o	o	o	o	o	o	o	o	o	o

Q.2 2.

	Least relevant 1 (1)	2 (2)	3 (3)	4 (4)	5 (5)	6 (6)	7 (7)	8 (8)	9 (9)	Most relevant 10 (10)
Improved overall population health (2)	o	o	o	o	o	o	o	o	o	o

Q.3 3.

	Least relevant 1 (1)	2 (2)	3 (3)	4 (4)	5 (5)	6 (6)	7 (7)	8 (8)	9 (9)	Most relevant 10 (10)
Saving money (3)	o	o	o	o	o	o	o	o	o	o

Q.4 4.

	Least relevant 1 (1)	2 (2)	3 (3)	4 (4)	5 (5)	6 (6)	7 (7)	8 (8)	9 (9)	Most relevant 10 (10)
Improved capability for improvement (e.g., new skills) (5)	o	o	o	o	o	o	o	o	o	o

Q.5 5.

	Least relevant 1 (1)	2 (2)	3 (3)	4 (4)	5 (5)	6 (6)	7 (7)	8 (8)	9 (9)	Most relevant 10 (10)
Increased capacity for improvement (e.g., more data collection resources) (4)	O	O	O	O	O	O	O	O	O	O

Q.6 6.

	Least relevant 1 (1)	2 (2)	3 (3)	4 (4)	5 (5)	6 (6)	7 (7)	8 (8)	9 (9)	Most relevant 10 (10)
Financial sustainability (6)	o	o	o	o	o	o	o	o	o	o

Q.7 7.

	Least relevant 1 (1)	2 (2)	3 (3)	4 (4)	5 (5)	6 (6)	7 (7)	8 (8)	9 (9)	Most relevant 10 (10)
Improved reputation for quality care (7)	o	o	o	o	o	o	o	o	o	o

Q. 8 8.

	Least relevant 1 (1)	2 (2)	3 (3)	4 (4)	5 (5)	6 (6)	7 (7)	8 (8)	9 (9)	Most relevant 10 (10)
Improved productivity (better use of resources) (8)	O	O	O	O	O	O	O	O	O	O

Q. 9 9.

	Least relevant 1 (1)	2 (2)	3 (3)	4 (4)	5 (5)	6 (6)	7 (7)	8 (8)	9 (9)	Most relevant 10 (10)
Improved efficiency (reducing waste) (9)	O	O	O	O	O	O	O	O	O	O

Q. 10 10.

	Least relevant 1 (1)	2 (2)	3 (3)	4 (4)	5 (5)	6 (6)	7 (7)	8 (8)	9 (9)	Most relevant 10 (10)
Generating revenue or income (10)	O	O	O	O	O	O	O	O	O	O

Q. 11 11.

	Least relevant 1 (1)	2 (2)	3 (3)	4 (4)	5 (5)	6 (6)	7 (7)	8 (8)	9 (9)	Most relevant 10 (10)
Improved service user access to care (11)	O	O	O	O	O	O	O	O	O	O

Q. 12 12.

	Least relevant 1 (1)	2 (2)	3 (3)	4 (4)	5 (5)	6 (6)	7 (7)	8 (8)	9 (9)	Most relevant 10 (10)
Improved staff outcomes e.g., wellbeing & retention (12)	o	o	o	o	o	o	o	o	o	o

Q. 13 13.

	Least relevant 1 (1)	2 (2)	3 (3)	4 (4)	5 (5)	6 (6)	7 (7)	8 (8)	9 (9)	Most relevant 10 (10)
Improved internal collaboration (19)	o	o	o	o	o	o	o	o	o	o

Q. 14 14.

	Least relevant 1 (1)	2 (2)	3 (3)	4 (4)	5 (5)	6 (6)	7 (7)	8 (8)	9 (9)	Most relevant 10 (10)
Development of a culture of providing quality care (15)	O	O	O	O	O	O	O	O	O	O

Q. 15 15.

	Least relevant 1 (1)	2 (2)	3 (3)	4 (4)	5 (5)	6 (6)	7 (7)	8 (8)	9 (9)	Most relevant 10 (10)
Improved status e.g., being a Foundation Trust (10)	O	O	O	O	O	O	O	O	O	O

Q. 16 16.

	Least relevant 1 (1)	2 (2)	3 (3)	4 (4)	5 (5)	6 (6)	7 (7)	8 (8)	9 (9)	Most relevant 10 (10)
Improved relationship with regulators e.g., CQC (17)	O	O	O	O	O	O	O	O	O	O

Q. 17 17.

	Least relevant 1 (1)	2 (2)	3 (3)	4 (4)	5 (5)	6 (6)	7 (7)	8 (8)	9 (9)	Most relevant 10 (10)
Improved service user outcomes e.g., experience and engagement (13)	o	o	o	o	o	o	o	o	o	o

Q. 18 18.

	Least relevant 1 (1)	2 (2)	3 (3)	4 (4)	5 (5)	6 (6)	7 (7)	8 (8)	9 (9)	Most relevant 10 (10)
Being the provider of choice (20)	o	o	o	o	o	o	o	o	o	o

Q. 19 19.

	Least relevant 1 (1)	2 (2)	3 (3)	4 (4)	5 (5)	6 (6)	7 (7)	8 (8)	9 (9)	Most relevant 10 (10)
Developing research skills (20)	o	o	o	o	o	o	o	o	o	o

Q. 20 20.

	Least relevant 1 (1)	2 (2)	3 (3)	4 (4)	5 (5)	6 (6)	7 (7)	8 (8)	9 (9)	Most relevant 10 (10)
Helping an organisation become innovative (29)	O	O	O	O	O	O	O	O	O	O

Q. 21 21.

	Least relevant 1 (1)	2 (2)	3 (3)	4 (4)	5 (5)	6 (6)	7 (7)	8 (8)	9 (9)	Most relevant 10 (10)
Organisational sustainability (22)	O	O	O	O	O	O	O	O	O	O

Q. 22 22.

	Least relevant 1 (1)	2 (2)	3 (3)	4 (4)	5 (5)	6 (6)	7 (7)	8 (8)	9 (9)	Most relevant 10 (10)
Minimising need for costly care (3)	O	O	O	O	O	O	O	O	O	O

Q.23 23.

	Least relevant 1 (1)	2 (2)	3 (3)	4 (4)	5 (5)	6 (6)	7 (7)	8 (8)	9 (9)	Most relevant 10 (10)
Generating profit (29)	O	O	O	O	O	O	O	O	O	O

Q. 24 24.

	Least relevant 1 (1)	2 (2)	3 (3)	4 (4)	5 (5)	6 (6)	7 (7)	8 (8)	9 (9)	Most relevant 10 (10)
Being better than competitors (3)	O	O	O	O	O	O	O	O	O	O

Q. 25 25.

	Least relevant 1 (1)	2 (2)	3 (3)	4 (4)	5 (5)	6 (6)	7 (7)	8 (8)	9 (9)	Most relevant 10 (10)
Improved collaboration with external partners (3)	O	O	O	O	O	O	O	O	O	O

Q. 26 26.

	Least relevant 1 (1)	2 (2)	3 (3)	4 (4)	5 (5)	6 (6)	7 (7)	8 (8)	9 (9)	Most relevant 10 (10)
Preventing mental health crises (3)	O	O	O	O	O	O	O	O	O	O

COMMENTS OPTIONAL COMMENTS

Please place an optional comment regarding your scores above.

____

End of Block: SECTION 1

Start of Block: SECTION 2

Section B SECTION B: AGREE/DISAGREE

My previous studies indicated some differences of opinion on some aspects of return on investment from QI programmes.

Please indicate if you agree or disagree with each statement below.

____

Q.27-32 Q. 27-32 INTERVENTION SUCCESS

Definition

Linked to the design and goals of a programme.

	Strongly disagree 1 (1)	Disagree 2 (2)	Somewhat disagree 3 (3)	Neither agree nor disagree 4 (4)	Somewhat agree 5 (5)	Agree 6 (6)	Strongly agree 7 (7)
27. Only benefits that are related to a programmes intended goals represent return on investment. (33)	o	o	o	o	o	o	o
28. Benefits beyond intended goals are valid returns on investment (34)	o	o	o	o	o	o	o
29. Lessons learnt even when intended goals were not achieved are a valid return on investment. (35)	o	o	o	o	o	o	o

30. A legacy left by QI (e.g., better awareness and capabilities) is a valid return on investment. (39)	O	O	O	O	O	O	O
31. QI is trial and error and therefore it cannot fail. (36)	O	O	O	O	O	O	O
32. A QI programme has failed if it has not achieved its intended goals. (47)	O	O	O	O	O	O	O

33-37 Q. 33-37 IMPLEMENTATION SUCCESS

Definition

Linked to the design and process of implementation.

	Strongly disagree 1 (1)	Disagree 2 (2)	Somewhat disagree 3 (3)	Neither agree nor disagree 4 (4)	Somewhat agree 5 (5)	Agree 6 (6)	Strongly agree 7 (7)
33. A QI programme has failed if the new practice has not spread in an organisation. (28)	O	O	O	O	O	O	O
34. A QI programme has failed if the new practice has not been embedded in an organisation. (29)	O	O	O	O	O	O	O
35. A QI programme has failed if improvements from a programme have not been sustained. (30)	O	O	O	O	O	O	O

36. When QI is embedded, it is easier and faster to pick up and solve quality problems. (32)	o	o	o	o	o	o	o
37. Being faster at picking up and solving quality problems is a sign of QI return on investment. (31)	o	o	o	o	o	o	o

Q.38-40 Q. 38-40 SHORT-TERM and LONG-TERM OUTCOMES

	Strongly disagree 1 (1)	Disagree 2 (2)	Somewhat disagree 3 (3)	Neither agree nor disagree 4 (4)	Somewhat agree 5 (5)	Agree 6 (6)	Strongly agree 7 (7)
38. Only benefits that occur during a programme implementation should be part of return on investment. (1)	O	O	O	O	O	O	O
39. Only benefits occurring months and years after a programme ends should be included. (2)	O	O	O	O	O	O	O
40. Both benefits during and after a programme ends should be included. (3)	O	O	O	O	O	O	O

41-44 Q. 41-44 EXTERNAL OUTCOMES

Definition

External: outside a registered healthcare entity with unique accountability.

	Strongly disagree 1 (1)	Disagree 2 (2)	Somewhat disagree 3 (3)	Neither agree nor disagree 4 (4)	Somewhat agree 5 (5)	Agree 6 (6)	Strongly agree 7 (7)
41. Service users' socio-economic benefits are part of an organisation's QI return on investment. (4)	o	o	o	o	o	o	o
42. Benefits to carers, family & friends are part of QI return on investment. (5)	o	o	o	o	o	o	o
43. Benefits to external partners are part of QI return on investment. (6)	o	o	o	o	o	o	o

44. Benefits to communities and societies are part of QI return on investment. (37)	O	O	O	O	O	O	O

45-48 Q. 45-48 MEASURABILITY

Definition

Ability to quantify benefits.

	Strongly disagree 1 (1)	Disagree 2 (2)	Somewhat disagree 3 (3)	Neither agree nor disagree 4 (4)	Somewhat agree 5 (5)	Agree 6 (6)	Strongly agree 7 (7)
45. Only measurable benefits represent QI return on investment. (7)	O	O	O	O	O	O	O
46. Immeasurable benefits are equally valid as returns on investment. (14)	O	O	O	O	O	O	O
47. Immeasurable benefits are more valid as returns on investment. (15)	O	O	O	O	O	O	O
48. Immeasurable benefits are sometimes more valid as QI returns on investment. (13)	O	O	O	O	O	O	O

Q. 49-57 Q. 49-57 MONETISABILITY

Definition Ability to quantify and convert benefits to money

	Strongly disagree 1 (1)	Disagree 2 (2)	Somewhat disagree 3 (3)	Neither agree nor disagree 4 (4)	Somewhat agree 5 (5)	Agree 6 (6)	Strongly agree 7 (7)
49. Only monetised benefits represent QI return on investment. (16)	o	o	o	o	o	o	o
50. Difficult to monetise benefits e.g., wellbeing are as equally valid as monetisable benefits. (17)	o	o	o	o	o	o	o
51. Difficult to monetise benefits are more important than monetisable benefits. (19)	o	o	o	o	o	o	o

52. Difficult to monetise benefits are sometimes more important than monetisable benefits. (18)	O	O	O	O	O	O	O
53. Monetisation of QI outcomes is valid because it is the stipulated requirement for evidence of QI value. (45)	O	O	O	O	O	O	O
54. Monetisation of QI outcomes is valid because it is best practice for evidence of QI value. (46)	O	O	O	O	O	O	O

55. Monetising benefits is impractical, I think there should be an alternative way to assess QI value. (47)	O	O	O	O	O	O	O
56. Monetisation is against my professional values, there should be an alternative way to assess QI value. (23)	O	O	O	O	O	O	O
57. Monetisation is against mental healthcare values, there should be an alternative way to assess QI value. (24)	O	O	O	O	O	O	O

Q. 58 Q. 58 ATTRIBUTION (i)

Definition
Ability to directly link cause (QI) to effect (benefit).

	Strongly disagree 1 (1)	Disagree 2 (2)	Somewhat disagree 3 (3)	Neither agree nor disagree 4 (4)	Somewhat agree 5 (5)	Agree 6 (6)	Strongly agree 7 (7)
58. Only benefits that can be linked directly to a QI programme count as returns on investment. (25)	O	O	O	O	O	O	O

Q.59-64 Q. 59-64 ATTRIBUTION (ii)

Definition **Indicator:** A measurable change that shows progress towards or achievement of a desired output or outcome.

	Strongly disagree 1 (1)	Disagree 2 (2)	Somewhat disagree 3 (3)	Neither agree nor disagree 4 (4)	Somewhat agree 5 (5)	Agree 6 (6)	Strongly agree 7 (7)
59. Valid indicators of hard to measure benefits (e.g., experience) are adequate evidence of return on investment. (8)	O	O	O	O	O	O	O
60. A narrative report of difficult to measure benefits in addition to measured outcomes is adequate evidence of return on investment. (9)	O	O	O	O	O	O	O

61. Subjective judgement of benefit without measurement is acceptable. (21)	O	O	O	O	O	O	O
62. Subjective judgement about benefits is acceptable provided there are agreed criteria. (10)	O	O	O	O	O	O	O
63. Subjective judgement criteria must be agreed specific to each Trust. (11)	O	O	O	O	O	O	O
64. Common subjective criteria should apply across all mental healthcare Trusts. (12)	O	O	O	O	O	O	O

Q.65-67 Q. 65-67 ATTRIBUTION (iii)

Definition **Proxy:** A financial value estimate of a benefit that has no market (financial) value e.g., patient experience.

	Strongly disagree 1 (1)	Disagree 2 (2)	Somewhat disagree 3 (3)	Neither agree nor disagree 4 (4)	Somewhat agree 5 (5)	Agree 6 (6)	Strongly agree 7 (7)
65. Proxies of difficult to monetise benefits are acceptable evidence of return on investment. (20)	O	O	O	O	O	O	O
66. A narrative report of difficult to monetise benefits in addition to monetised outcomes is acceptable. (22)	O	O	O	O	O	O	O

67. Subjective judgement about benefits is acceptable provided there are agreed criteria. (10)	O	O	O	O	O	O	O

OPTIONAL TEXT OPTIONAL COMMENTS

Please share any relevant considerations about QI-ROI you think are missing above.

____

End of Block: SECTION 2

Start of Block: SECTION 3

DEMOGRAPHICS What best describes your role and background as a mental healthcare leader. Please select maximum 2 options.

- ▢ Executive board member: economics background (1)
- ▢ Executive board member: no economics background (2)
- ▢ Non-executive board member: economics background (3)
- ▢ Non-executive board member: no economics background (4)
- ▢ Director: economics background (5)
- ▢ Director: no economics background (6)
- ▢ Quality Improvement Leadership: economics background (7)
- ▢ Quality Improvement Leadership: no economics background (8)

End of Block: SECTION 3

Supplementary file 3: Example Round 2 feedback format

TITLE OF PROJECT:

The development of the QI-ROI conceptual framework for mental health; a DELPHI study. Ethical review reference: King’s College London; MRSP-22/23-33873

ROUND 2

-------------------------------------**-------------------------------------

Thank you for taking part in this SECOND ROUND of the study

This round is to ascertain if there has been any change in some opinions since the last round. In this round, [we] hope to gain even more clarity on the components of QI benefits that are seen to represent ROI. More clarity will help provide a more concise definition of QI-ROI and further develop the QI-ROI conceptual framework as it stands currently. As such, [we] have asked for some statements to be re-rated.

Only these statements are to be re-rated:

- statements that did not achieve agreement
- neutral responses
- outlying responses

Only statements that require you attention will be re-sent to you for re-rating. Each statement will be provided with feedback from the last round. All feedback and study reports are anonymised. You can keep your previous response or change as you wish. If unchanged, [we] would appreciate any insights you may wish to share regarding your rating. This may be helpful as insights to feedback to other participants and for further exploration in future studies.

Should you with to share any new information about other or statements from the previous round, please use the optional comment space provided at the end. This may also be helpful as insights to feedback to other participants and for further exploration in future studies.

Many thanks in advance.

Example: (*R1, R2=rounds one and two in the first two boxplots in the graphs below*)

As can be seen in the attached graph, in the PREVIOUS ROUND,

- the median response was 2 on a scale of 7.
- This implied that most participants disagree with the given statement that

“Only benefits that occur during a programme implementation should be part of ROI”.

Do you agree/disagree with the median and its implications?
Please **RE-RATE** this statement to either confirm or change your rating.
Please **leave an optional note below this sentence** to support or explain your choice.

All re-rated statements to be introduced this way; participants blinded to their round 1 score. Only group medians and an accompanying graph to be provided to all participants, i.e., no individual scores to be returned to individuals

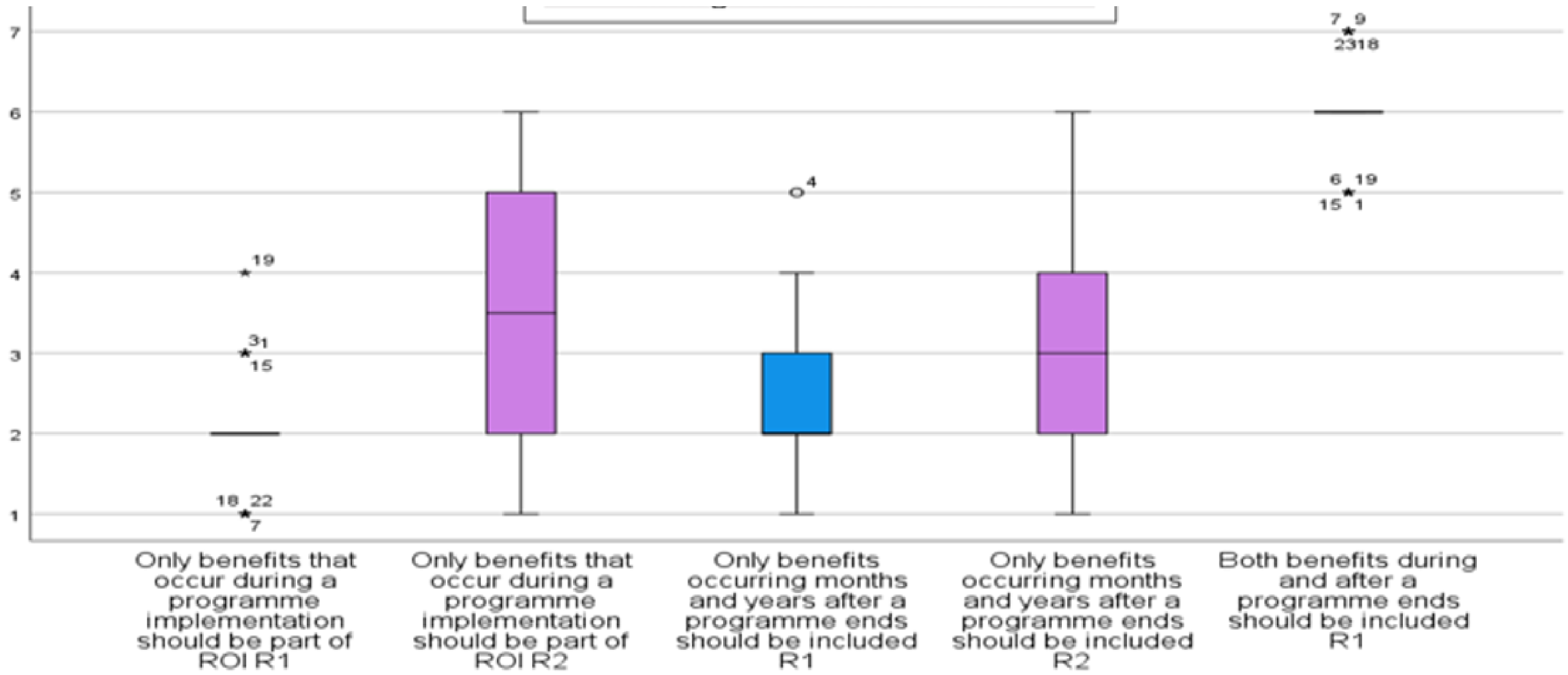

Legend: In these graphs, the blue and single line items were rated in round one, and the purple items were re-rated. Figure 6.8 was borrowed from [first author] PhD thesis.

Supplementary file 4: Summative results

SECTION A<br>Item relevance 1-10 # RIR (relative interquartile range) >30% unstable # IQR > 3 not accepted as consensus # Outliers cancel IQR consensus												
Item	Min Score R1(R2)	Max Score R1(R2)	Median		IQR		RIR		RIR variation	Consensus<br>*Outliers<br>Green-consensus<br>Orange-undecided<br>Red-dissensus	Stability
			R	R2	R1	R2	RI	R2			
1.Health outcomes	5	10	10	-	2	-	-			Strong consensus	-
2.Population health	4	10	8	-	2	-	-			Strong consensus	-
3.Cost saving	2 (2)	10 (8)	6	6	3	2	0.5	0.3	0.2	Undecided	Stable
4.Capability development	5	10	8	-	3	-	-			Moderate consensus	-
5.Capacity development	3	10	7	-	3	-	-			Moderate consensus	-
6.Financial sustainability	4	10	8	-	3	-	-			Moderate consensus	-
7.Reputation	4	10	8	-	3	-	-			Moderate consensus	-
8.Productivity	5	10	8	-	1.5		-			Strong consensus	-
9.Efficiency	4	9	8	-	2	-	-			Strong consensus	-
10.Revenue generation	1 (1)	9 (7)	5	4	3.5	3	0.7	0.75	0	Moderate dissensus	Stable
11.Care access	4 (5)	10 (10)	8	8	3	2	0.38	0.25	0.13	Strong consensus	Stable
12.Staff outcomes	5	10	8	-	2	-	-			Strong consensus	-

13.Internal collaboration	5	9	7	-	2	-	-			Strong consensus	-
14.Quality and safety culture	4	10	9	-	2	-	-			Strong consensus	-
15.Registration status	1 (1)	9 (6)	5	4	4	3	0.8	0.75	0	Moderate dissensus	Stable
16.Oversight benefits e.g., improved CQC rating	1 (2)	9 (10)	6	7	3	3	0.5	0.43	0	Moderate consensus	Stable
17.Patient experience	4	10	9	-	3	-	-			Strong consensus	-
18.Provider of choice	1 (1)	10 (10)	5	6.5	4	5	0.8	0.77	0	Very high dissensus	Stable
19.Research development	1 (2)	9 (10)	6	6	2.5	2	0.42	0.33	0	Very high dissensus*	Stable
20.Innovation development	3	9	7	-	2	-	-			Strong consensus	-
21.Organisational sustainability	5	10	8	-	2	-	-			Strong consensus	-
22.Avoiding costly care	2	10	7	-	3	-	-			Moderate consensus	-
23.Profit generation	1 (1)	10 (7)	3	3	3	3	1	1	0	Moderate consensus -ve	Stable
24.Competitiveness	1 (1)	9 (9)	4	5.5	6	4	1.5	0.72	0.78	Very high dissensus	Unstable
25.External collaboration	2 (2)	9 (10)	7	7	1.5	2	0.2	0.29	0	Very high dissensus*	
26.Preventing mental health crises	1 (2)	10 (10)	8	7.5	4	4	0.5	0.53	0	Moderate dissensus	Stable
**SECTION B**<br>**Item eligibility: 1 -7 # RIR (relative interquartile range) >30% unstable # IQR > 2 not accepted as consensus # Outliers cancels IQR consensus**											
Item	Min Score R1(R2)	Max Score R1(R2)	Median		IQR		RIR		RIR variation	Consensus *outliers	Stability
			R1	R2	R1	R2	R1	R2			
27.Goal achievement	1 (1)	6 (5)	2	2	0.5	0.5	0.25	0.25	0	Very high dissensus*	Stable
28.Benefits beyond goals (e.g., unintended developments)	5	7	6	-	0	-	-			Very strong consensus	-

29.Lessons learnt (e.g., from failed QI)	2 (4)	7 (7)	6	6	1	1	0.17	0.17	0	Ver strong consensus	Stable
30.QI legacy (e.g., raised safety awareness)	2 (2)	7 (7)	5	6	1	1	0.2	0.17	0	Very high dissensus*	Stable
31.QI cannot fail	1 (1)	6 (1)	3	3	1.5	2	0.5	0.67	0	Very high dissensus*	Stable
32.QI failed if not goals not achieved	1 (1)	6 (5)	3	3	1	3	0.3	1	0.7	Moderate dissensus	Unstable
33. QI failed if not programme not spread	1 (2)	6 (6)	3	4	2.5	3	0.8	0.75	0	Moderate dissensus	Stable
34. QI failed if not new practice not embedded	1 (2)	6 (6)	5	5	1.5	4	0.3	0.8	0.5	High dissensus	Unstable
35. QI failed if not programme/benefits not sustained	1 (2)	6 (6)	5	5	0.5	2	0.1	0.4	0.3	Very high dissensus	Stable
36.Problem solving/programme speed is a sign of QI embedment	3 (4)	7 (7)	5	6	1	1	0.2	0.17	0	Very strong consensus	Stable
37. Problem solving/programme speed is an indicator of QI-ROI	4	7	5	-	1	-	-			Very strong consensus	-
38.Short-term outcomes	1 (1)	4 (6)	2	3	0	3	0	1	1	Moderate dissensus	Unstable
39.Long-term outcomes	1 (1)	5 (6)	2	3	1	2	0.5	0.67	0.17	Strong consensus -ve	Stable
40.Both short and long-term outcomes are	5	7	6	-	0	-	-			Very strong consensus	-
41.Service-user socio-economic benefits	2 (3)	7 (6)	5	5	1	1	0.2	0.2	0	Very high dissensus*	Stable
42.Friends, families, carers' benefits	4	7	6	-	2	-	-			Strong consensus	-
43.External partners benefits	4	7	6	-	1	-	-			Very strong consensus	-
44.Community and societal benefits	5	7	6	-	0.5	-	-			Very strong consensus	-
45.Only measurable benefits are ROI	1	6	3	-	3	-	-			Moderate dissensus	-
46.Immeasurable benefits are equally valid ROI	2	7	5	-	2	-	-			Strong consensus	-
47.Immeasurable benefits are more valid as ROI	1	5	3	-	2	-	-			Strong consensus -ve	-
48.Immeasurable benefits are sometimes more valid as ROI	2 (3)	7 (7)	4	5	2	2	0.5	0.4	0.1	Strong consensus	Stable
49.Only monetisable benefits are ROI	1	6	2	-	2	-	-			Strong consensus -ve	-

50.Difficult to monetise benefits are equally valid as ROI	4	7	5	-	1	-	-			Very strong consensus	-
51.Difficult to monetise benefits are more important	1	5	4	-	2	-	-			Undecided	-
52.Difficult to monetise benefits are sometimes more important	2 (2)	7 (7)	5	5.25	1	1	0.2	0.19	0	Strong consensus	Stable
53.Monetisation is valid as it is the stipulated requirement	1	6	4	-	1.5	-	-			Undecided	-
54.Monetisation is valid as it is the best practice	1	6	4	-	2	-	-			Undecided	-
55.Monetisation is impractical, there should be an alternative	2	7	4	-	2	-	-			Undecided	-
56.Monetisation is against professional values	1	5	2	-	1.5	-	-			Strong consensus	-
57.Monetisation is against mental healthcare values	1	5	2	-	2	-	-			Strong consensus	-
58.Only benefits that be directly linked to a programme are ROI	1	6	3	-	3	-	-			Moderate dissensus	-
59.Valid indicators of hard to measure benefits are valid evidence	3 (3)	7 (7)	5	5.5	1	1	0.2	0.18	0	Very high dissensus*	Stable
60.A narrative report of benefits is valid evidence for ROI	3 (2)	7 (7)	5	6	1	2	0.2	0.33	0.13	Strong consensus	Stable
61.Subjective judgement of benefit measurement is acceptable	2	6	3	-	2	-	-			Strong consensus -ve	-
62.Subjective judgement is valid evidence of ROI if criteria agreed	2 (2)	6 (6)	5	5	1	3	0.2	0.6	0.4	Moderate dissensus	Unstable
63.Subjective criteria should be decided per Trust	2	6	4	-	1.5	-	-			Undecided	-
64.Subjective criteria should apply across mental healthcare Trusts	2	6	4	-	2	-	-			Undecided	-
65.Financial proxies are acceptable as ROI evidence	3 (3)	7 (6)	5	5	0.5	1	0.1	0.2	0.1	Very high dissensus*	Stable
66.A narrative report of difficult to monetise benefits is acceptable	3 (2)	7 (7)	5	6	1	1	0.2	0.17	0	Very high dissensus*	Stable
67.Subjective judgement about monetary benefit is valid evidence	3	6	5	-	1	-	-			Strong consensus	-
